# Psychometric properties of the self-report version of the Quick Inventory of Depressive Symptoms (QIDS-SR_16_) questionnaire in patients with schizophrenia

**DOI:** 10.1186/s12888-014-0247-2

**Published:** 2014-09-03

**Authors:** Irene M Lako, Johanna TW Wigman, Rianne MC Klaassen, Cees J Slooff, Katja Taxis, Agna A Bartels-Velthuis, GROUP investigators

**Affiliations:** Division of Pharmacotherapy and Pharmaceutical Care, Department of Pharmacy, University of Groningen, Groningen, The Netherlands; University of Groningen, University Medical Center Groningen, University Center for Psychiatry, Rob Giel Research Center, Groningen, The Netherlands; Department of Psychiatry and Psychology, Maastricht University Medical Center, Maastricht, The Netherlands; Rivierduinen Mental Health, Leiden, The Netherlands; Department of Psychotic Disorders, Mental Health Center Assen (GGZ Drenthe), Assen, Netherlands

## Abstract

**Background:**

Self-report instruments for the assessment of depressive symptoms in patients with psychotic disorders are scarce. The Quick Inventory of Depressive Symptoms (QIDS-SR_16_) may be a useful self-report instrument, but has received little attention in this field. This paper aimed to test the psychometric properties of the QIDS-SR_16_ questionnaire in patients with a psychotic disorder.

**Methods:**

Patients diagnosed with a psychotic disorder from health care institutions in The Netherlands were included in the study. Depressive symptoms were assessed with the QIDS-SR_16_ and the Calgary Depression Scale for Schizophrenia (CDSS). Psychotic symptoms were assessed with the Positive and Negative Syndrome Scale (PANSS) and extrapyramidal symptoms (EPS) with three EPS rating scales. Spearman’s correlation coefficients were used to compare the total score of the QIDS-SR_16_ with the total scores of the CDSS, PANSS-subscales and EPS rating scales.

**Results:**

In a sample of 621 patients with psychotic disorders, the QIDS-SR_16_ showed good internal consistency (α = 0.87). The QIDS-SR_16_ correlated moderately with the CDSS (r = 0.44) and the PANSS subscale for emotional distress (r = 0.47). The QIDS-SR_16_ showed weak correlation with the PANSS subscale for negative symptoms (r = 0.28) and minimal correlation with EPS rating scales (r = 0.09-0.16).

**Conclusions:**

The QIDS-SR_16_ may reliably assess depressive symptoms in patients with psychotic disorders, but its concurrent validity with the CDSS was rather poor in this population. We would recommend developing a new self-report questionnaire for the assessment of depressive symptoms in patients with psychotic disorders.

**Electronic supplementary material:**

The online version of this article (doi:10.1186/s12888-014-0247-2) contains supplementary material, which is available to authorized users.

## Background

Depressive symptoms are highly prevalent in patients with schizophrenia, with prevalence rates estimated between 7% and 75% [1,2]. Depressive symptoms are present throughout all phases of the illness [3] and may lead to a higher burden of disease and more frequent relapses [4,5]. Screening and routine monitoring of these symptoms may guide appropriate treatment [6,7]. Depressive symptoms can be difficult to distinguish from negative symptoms and extrapyramidal symptoms (EPS), such as drug-induced parkinsonism [8]. Adequate recognition of depressive symptoms, as well as regular monitoring of symptomatic changes is essential to guide appropriate treatment in patients with schizophrenia [7,9]. Therefore, monitoring depressive symptoms requires reliable instruments with tested validity in patients with schizophrenia. To date, the only instrument designed for the assessment of depressive symptoms in this patient population is the interview-based Calgary Depression Scale for Schizophrenia (CDSS) [10]. The CDSS is a reliable and valid instrument that is able to distinguish depressive symptoms from negative psychotic symptoms and EPS [10]. However, the interview-based assessment method has some drawbacks, such as the need for trained interviewers and observer bias. Self-report may be as good as interview-based assessments for monitoring change in psychopathology [11] and saves time and costs in routine clinical practice [12]. The availability of self-report depression instruments with comparable reliability and validity in patients with schizophrenia is however limited [13]. The Beck Depression Inventory-II (BDI) is the only self-report depression instrument for which complete information on psychometric properties in a population with schizophrenia are available for review [14]. Review of these properties demonstrated that the concurrent and predictive validity of the BDI was rather poor, perhaps because almost half of the items of the BDI could also be interpreted as negative symptoms [13].

The Quick Inventory of Depressive Symptoms (QIDS-SR_16_) is a short and easy-to-use self-report instrument to assess depressive symptoms [15]. The QIDS-SR_16_ is sensitive to symptomatic change and its psychometric properties are good in patients with depressive disorders [16]. Furthermore, it was found that the presence of psychotic symptoms did not meaningfully affect the ability of self-rating to recognize depressive symptoms in patients with major depressive disorder [17]. To our knowledge, the reliability and validity of the QIDS-SR_16_ has not been tested in patients with schizophrenia. A question of specific interest is whether the QIDS-SR_16_ can distinguish depressive symptoms from negative and extrapyramidal symptoms in this population (divergent validity). Furthermore, it is unknown whether the latent structure of the QIDS-SR_16_ remains one-dimensional [18,19], or that multiple (negative symptom) dimensions can be identified when applied in patients with schizophrenia.

The aim of the current study is to evaluate the psychometric properties of the QIDS-SR_16_ in a population of patients with psychotic disorders. We examined (1) the internal consistency of the QIDS-SR_16_, (2) the dimensional structure, (3) the concurrent validity with other depression instruments and (4) the divergent validity with negative and extrapyramidal symptoms.

## Methods

### Subjects

Subjects were patients participating in the Genetic Risk and Outcome of Psychosis (GROUP) study, a naturalistic longitudinal cohort study. The longitudinal GROUP study is conducted by four academic centers in the Netherlands and a large number of mental health institutes in the Netherlands and the Dutch speaking region of Belgium. The GROUP study was approved centrally by the Ethical Review Board of the University Medical Center Utrecht and all participants gave written informed consent in accordance with the committee’s guidelines. For a detailed overview of the GROUP structure, data flow, quality control, recruitment, sample characteristics of the studied patients and training procedures of the assessors see Korver et al. [20]. The current data was collected during the second assessment of the study, three years after the baseline assessment (GROUP data release 3.02). Patients were included in the current study if they had a diagnosis of a psychotic disorder according to the Diagnostic and Statistical Manual of Mental Disorders (DSM-IV) criteria [21] and if data of the following rating scales were complete: the QIDS-SR_16_, CDSS, and Positive and Negative Syndrome Scale (PANSS) [22], Abnormal and Involuntary Movements Scale (AIMS) [23] and Barnes Akathisia Rating Scale (BARS) [24]. These rating scales were administered by trained research assistants.

All research assistants were very well trained in administering the instruments. Data on interrater reliability of the GROUP study were not yet available for the second assessment, but the intraclass correlation coefficient of PANSS total score of the first assessment was 0.946 (95% confidence interval 0.758 to 0.996) [20].

Two weeks before the assessment patients were sent the self-report questionnaires (i.c., QIDS-SR_16_)_,_ with the request to bring them along completed to the assessment. Interviews and tests were administered in a fixed order (i.c., PANSS, CDSS, EPS scales), normally on the same day.

### Measures

Patients completed the self-report version of QIDS-SR_16_ to assess depressive symptoms [15] (see for English version in Additional file 1, for multiple translations and scoring instructions see http://www.ids-qids.org/). The measure consists of 16 items, covering nine depressive symptom domains. Each domain score is based on the highest score on the pertaining items. Domain scores and item scores are rated on a Likert scale ranging from 0 to 3, with a total score range of 0–27. For an interpretation the QIDS-SR_16_ total score see http://www.ids-qids.org/. Depressive symptoms were also assessed by the 9-item CDSS interview [10]. Item scores are rated on a Likert scale ranging from 0 to 3. A sum score above 4 out of 27 on the CDSS was used as cut-off scores to establish the presence of a minor depressive episode or clinical depression [10,25]. Psychotic symptoms were assessed with the PANSS [22,26]. For the current analyses, we used the five-factor model of the PANSS [27], consisting of the subscales ‘positive symptoms’ , ‘negative symptoms’ , ‘disorganization symptoms’ , ‘excitement’ and ‘emotional distress’. Item scores of the PANSS range from 1 (not present) to 7 (extreme) and the subscales scores for negative symptoms range from 7–49 and 7-28 for emotional distress. Extrapyramidal symptoms were assessed using the AIMS [23], the BARS [24] and, when available, the ‘motor examination’ subscale of the Unified Parkinson's Disease Rating Scale (UPDRS) [28]. The CDSS interview, the PANSS interview and the EPS rating scales were administered by the same research assistant on the very same day. The self-report QIDS-SR_16_ was sent to the participant about two weeks prior to the assessment, with the request to fill in the questions and bring the questionnaire along to the research assistant. Different rating scales were used for the assessment EPS because each of the rating scales reflects a different subset of motor symptoms. The AIMS is focused on dyskinesia (involuntary movements), the BARS on akathisia (restlessness) and the UPDRS on parkinsonism. The symptoms measured by these scales may relate themselves differently to depressive symptoms. For example, depressive symptoms have also been associated with parkinsonism [8] and restlessness or psychomotor agitation is also a depressive symptoms (see question 16 of the QIDS-SR_16_).

### Statistical analyses

Psychometric properties of the QIDS-SR_16_ were examined using SPSS, version 16.0, and R (v.3.0.1) running in R-studio. The internal consistency of the QIDS-SR_16_ was assessed by calculating ordinal alpha, the conceptual equivalent to Cronbach’s alpha for ordinal data [29] with the R packages ‘psych’ [30], and ‘GPArotation’ [31]. A value of 0.80 or higher indicated good internal consistency [32]. Additionally, polychoric inter-item correlations of the QIDS-SR_16_ were calculated. Average values of r > 0.15 were deemed acceptable, since depressive symptoms as covered by the QIDS-SR_16_ may represent a broad construct [33]. The dimensional structure of the QIDS-SR_16_ was examined by using a parallel analysis to determine how many principal components should be extracted from the data (PCA) [34]. In parallel analysis, the factors are retained as long as the *i*th eigenvalue from the actual data is greater than the *i*th eigenvalue extracted from a randomly drawn dataset that is similar to the actual dataset in its number of cases and variables. The parallel analysis was based on the polychoric inter-item correlations and conducted with the R-package ‘psych’ [30]. If a 1-component structure was found, this would suggest that the items that are covered by the QIDS-SR_16_ are best represented by one underlying construct, i.e. depression. The total score of the QIDS-SR_16_ was compared with the scores on the CDSS, PANSS and EPS rating scales. Concurrent validity was investigated by calculating Spearman correlations *(ρ)* of the QIDS-SR_16_ with the CDSS and the PANSS subscale for emotional distress. Divergent validity was examined by calculating Spearman correlations of the QIDS-SR_16_ with the PANSS-Negative symptoms subscale and the three EPS rating scales. Spearman correlations were used because of non-normality of the data. Bootstrapping was used to calculate the 95% confidence intervals (95% CI) of the correlations.

## Results

### Sample

Overall, 809 (72%) of the 1119 patients with a psychotic disorder who presented at baseline participated in the second assessment. Patients who participated in the second assessment did not differ in age (F[1,1117] = 3.15; *p* = 0.076), gender (*χ*^*2*^[1] = 0.71; *p* = 0.40) or duration of illness (F[1,1032] = 3.26; *p* = 0.071) from those who only completed baseline assessment. Of the 809 patients who participated in the second assessment, 621 patients completed all questionnaires that were required for inclusion in the current study (QIDS-SR_16_, CDSS and PANSS). Demographic and clinical descriptive information of this sample can be found in Table 1. The mean scores on the individual items of the QIDS-SR_16_ are given in Table 2. According to the CDSS, clinical depression was present among 17% (N = 103) of the patients.

**Table 1:** **Patient characteristics (N = 621)**

	**Mean (SD; range) or N (%)**
Age	30.1 (7.3; 18–59)
Male (%)	478 (77%)
Education	
Primary school	39 (6%)
Secondary school/high school	322 (52%)
Vocational education	150 (24%)
Vocational higher education	65 (11%)
University	45 (7%)
Illness duration (years)	7.3 (4.1; 2.0-43.1)
Age of onset first psychosis (years)	22.3 (6.7; 5–51)
Primary diagnosis	
Schizophrenia	398 (64%)
Schizoaffective disorder	80 (13%)
Schizophreniform disorder	37 (6%)
Delusional disorder	14 (2%)
Brief psychotic disorder	13 (2%)
Psychotic disorder NOS	64 (10%)
Other psychotic disorder	15 (2%)
Antidepressants^a^	81 (17%)
Antipsychotics^a^	
No antipsychotics	67 (14%)
Risperidone	58 (12%)
Olanzapine	91 (19%)
Quetiapine	28 (6%)
Clozapine	71 (15%)
Haloperidol	16 (3%)
Aripiprazol	50 (10%)
Other antipsychotics	35 (7%)
Combination therapy	65 (14%)
QIDS-SR_16_ (total)	6.6 (4.9; 0–26)
CDSS (total)	2.0 (2.8; 1–16)
PANSS Total	61.8 (18.9; 41–148)
PANSS-EMO (emotional distress)	13.1 (4.8; 8–33)
PANSS-NEG (negative symptoms)	12.6 (5.4; 4–41)
PANSS-POS (positive symptoms)	11.3 (5.4; 3–39)
PANSS-DIS (disorganized symptoms)	14.2 (5.1; 10–46)
PANSS-EXC (excitement symptoms)	10.6 (3.2; 2–29)
AIMS (total)	0.1 (0.2; 0–1.9)
BARS (total)	0.3 (0.6; 0–4.0)
UPDRS (subtotal motor symptoms)^b^	0.2 (0.1; 0–1.4)

*Abbreviations*: *SD* Standard Deviation; *QIDS-SR*_*16*_ Quick Inventory of Depressive Symptomatology 16-item self-report version; *CDSS* Calgary Depression Scale for Schizophrenia; *PANSS* Positive and Negative Syndrome Scale; *AIMS* Abnormal Involuntary Movement Scale; *BARS* Barnes Akathisia Rating Scale and *UPDRS* Unified Parkinson’s Disease Rating Scale.

^a^ Data on medication was available for n = 481 (77%) patients.

^b^ UPDRS ratings were available for n = 531 (85%) patients.

**Table 2:** **Mean scores on individual items of the QIDS-SR**_**16**_ **(N = 621)**

**QIDS-SR**_**16**_ **items**		**Mean**	**SD**
1	Sleep onset insomnia	0.93	1.09
2	Mid-nocturnal insomnia	0.76	0.99
3	Early morning insomnia	0.41	0.86
4	Hypersomnia (excessive sleep)	1.03	0.92
5	Feeling depressed	0.65	0.76
6	Decreased appetite	0.20	0.50
7	Increased appetite	0.35	0.69
8	Weight reduction	0.39	0.79
9	Weight gain	0.36	0.77
10	Concentration/decision making	0.64	0.81
11	Self-view	0.69	1.09
12	Suicide ideation	0.32	0.68
13	General interest	0.40	0.75
14	Energy level	0.55	0.77
15	Feeling slowed down	0.35	0.72
16	Feeling restless	0.49	0.82

*Abbreviations*: *SD* Standard Deviation.

### Internal consistency and dimensionality

**Figure 1:**
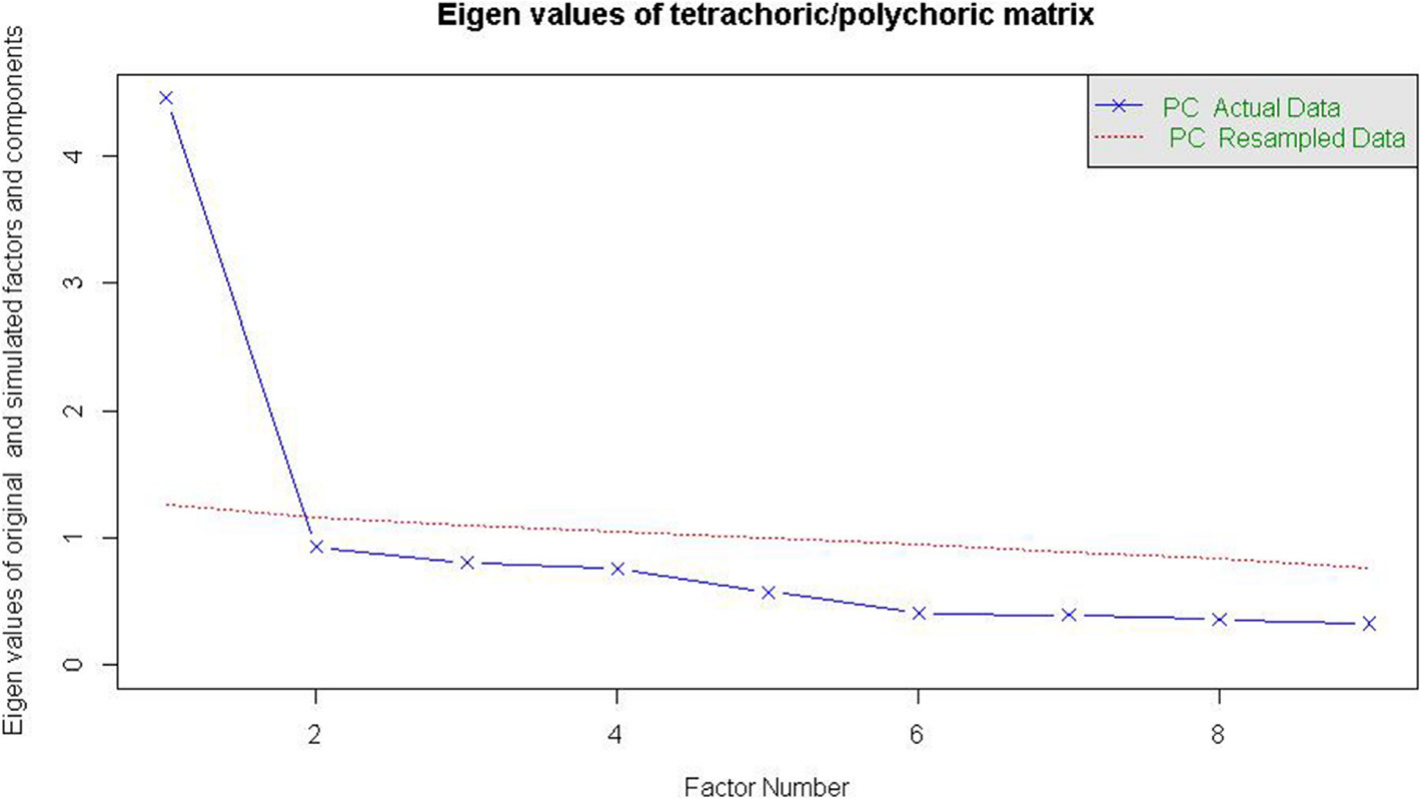
**Scree plot of obtained eigenvalues of the QIDS-SR**_**16**_ **, compared to eigenvalues based on random data.**

The QIDS-SR_16_ showed good internal consistency (ordinal alpha = 0.87). All individual inter-item correlations were within an acceptable range of 0.19-0.63 (Table 3) with an average inter-item correlation of 0.42. The parallel analysis results suggested that the data of the QIDS-SR_16_ can be reduced to one component in this sample (Figure 1).

**Table 3:** **Polychoric correlation coefficients (95%CI) between the individual items of the QIDS-SR**_**16**_

	**Sleep**	**Depressed mood**	**Appetite/weight**	**Concentration**	**Self-view**	**Suicidal ideation**	**Interest**	**Energy**	**Psychomotor**
Sleep	1								
Depressed mood	0.33 (0.23-0.42)	1							
Appetite/weight	0.19 (0.10-0.29)	0.22 (0.12-0.32)	1						
Concentration	0.33 (0.24-0.41)	0.47 (0.39-0.56)	0.37 (0.28-0.44)	1					
Self-view	0.36 (0.25-0.46)	0.58 (0.52-0.66)	0.29 (0.17-0.42)	0.52 (0.44-0.61)	1				
Suicidal ideation	0.36 (0.26-0.47)	0.59 (0.50-0.68)	0.30 (0.20-0.42)	0.39 (0.28-0.48)	0.61 (0.52-0.69)	1			
Interest	0.38 (0.27-0.48)	0.52 (0.42-0.61)	0.36 (0.26-0.45)	0.52 (0.42-0.61)	0.49 (0.37-0.59)	0.53 (0.42-0.63)	1		
Energy	0.39 (0.30-0.47)	0.44 (0.35-0.54)	0.29 (0.19-0.39)	0.54 (0.47-0.62)	0.42 (0.33-0.52)	0.35 (0.24-0.46)	0.56 (0.47-0.65)	1	
Psychomotor	0.24 (0.12-0.34)	0.46 (0.36-0.56)	0.35 (0.25-0.44)	0.63 (0.56-0.70)	0.52 (0.45-0.60)	0.38 (0.28-0.48)	0.46 (0.35-0.55)	0.47 (0.39-0.55)	1

*Abbreviations*: *QIDS-SR*_*16*_ Quick Inventory of Depressive Symptoms 16-item self-report version.

### Concurrent and divergent validity

The correlations of the individual domains of the QIDS-SR_16_ with the CDSS ranged between 0.14 and 0.46 (Table 4). The total score of the QIDS-SR_16_ correlated moderately with the CDSS (*ρ* = 0.44; *p* < .001) and the PANSS subscale for emotional distress (*ρ* = 0.47; *p* < .001), as displayed in Table 5. The QIDS-SR_16_ showed weaker correlations with negative symptom ratings of the PANSS (*ρ* = 0.28; *p* < .001) and extrapyramidal symptom ratings of the AIMS (*ρ* = 0.09; *p* < .05), BARS (*ρ* = 0.16; *p* < .001) and UPDRS-motor subscale (*ρ* = 0.13; *p* < .001).

**Table 4:** **Spearman correlations (95%CI) of QIDS-SR**_**16**_ **symptom domains with the CDSS total score**

**QIDS-SR**_**16**_ **domains**	**Correlation**
Sleep disturbance	0.22 (0.14-0.30)
Depressed (sad) mood	0.46 (0.38-0.52)
Change in appetite or weight	0.14 (0.07-0.22)
Concentration/decision making	0.27 (0.19-0.34)
Self-view	0.36 (0.28-0.43)
Suicidal ideation	0.38 (0.31-0.45)
Interest	0.33 (0.24-0.40)
Energy/fatigue	0.28 (0.21-0.35)
Psychomotor agitation/retardation	0.28 (0.20-0.35)

*Abbreviations*: *QIDS-SR*_*16*_ Quick Inventory of Depressive Symptoms 16-item self-report version; *CDSS* Calgary Depression Scale for Schizophrenia. All correlations were significant (*p* < .001).

**Table 5:** **Concurrent and divergent validity of the QIDS-SR**_**16**_ **total score**

		**QIDS-SR**_**16**_	**CDSS**	**PANSS-D**
Concurrent validity	QIDS-SR_16_	1		
	CDSS	0.44 (0.38-0.51)**	1	
	PANSS-D	0.47 (0.41-0.54)**	0.59 (0.54-0.64)**	1
Divergent validity	PANSS-N	0.28 (0.19-0.35)**	0.34 (0.28-0.41)**	0.40 (0.33-0.47)**
	AIMS	0.09 (0.01-0.16)*	0.06 (−0.01-0.14)	0.06 (−0.02-0.14)
	BARS	0.16 (0.09-0.24)**	0.09 (0.01-0.16)*	0.15 (0.07-0.23)**
	UPDRS-motor	0.13 (0.05-0.21)**	0.20 (0.13-0.28)**	0.25 (0.17-0.33)**

Concurrent validity of the QIDS-SR_16_ total score with other depression instruments and divergent validity with negative symptoms and extrapyramidal symptoms. Values are Spearman correlation coefficients (95% CI). Significant correlations were indicated by * = *p* < .05; ** = p < .001.

*Abbreviations*: *QIDS-SR*_*16*_ Quick Inventory of Depressive Symptoms 16-item self-report version; *CDSS* Calgary Depression Scale for Schizophrenia; *PANSS* Positive and Negative Syndrome Scale, emotional distress subscale (−D) and Negative symptom subscale (−N); *AIMS* Abnormal and Involuntary Movements Scale; *BARS* Barnes Akathisia Rating Scale and *UPDRS-motor*, Motor subscale of the Unified Parkinson’s Disease Rating Scale.

## Discussion

The current study was, to the best of our knowledge, the first to investigate the psychometric properties of the QIDS-SR_16_ in a large sample of patients with psychotic disorders. The QIDS-SR_16_ remained unidimensional in the current sample, representing depressive symptoms as an independent domain from negative symptoms and other psychotic symptoms in patients with schizophrenia [19,35]. Furthermore, the internal consistency of the QIDS-SR_16_ was good in our patient population, and comparable to that previously reported for the CDSS [36]. This suggests that patients with a psychotic disorder are able to rate their depressive symptoms in a reliable way [11]. The QIDS-SR_16_ agreed moderately with the CDSS, suggesting conceptual differences with the rating scale that is currently considered as the gold standard for assessment depressive symptoms in patients with schizophrenia.

These conceptual differences may reflect differences in item selection between the QIDS-SR_16_ and the CDSS. Unlike the CDSS, the QIDS-SR_16_ is not specifically designed to assess depressive symptoms in patients with psychotic disorders. Especially the QIDS-SR_16_ symptom domains on ‘sleep’ and ‘appetite' showed low agreement with the CDSS in our study. The scores on the sleep domain were relatively high compared to other domains of the QIDS-SR_16_; this was in most cases driven by the ‘hypersomnia’ item (excessive sleep) (see Table 2). Excessive sleep and increased appetite may reflect side effects of antipsychotics [37,38] and hence not necessarily be related to the ‘physical’ symptoms of depression [21]. Indeed, post hoc analysis using ordinal logistic regression demonstrated that those patients using antipsychotics with high antagonistic affinity for the histamine receptor (olanzapine or clozapine) reported higher scores on excessive sleep than patients using other antipsychotics (OR [95%CI] = 1.88 [1.31-2.68]). Similarly, patients using olanzapine or clozapine were more likely to report increased appetite (OR [95%CI] = 1.94 [1.28-2.95]). It can be argued that antipsychotic side effects confounded changes in sleep and appetite as measured by the QIDS-SR_16_ in the current sample. In contrast, the CDSS measures ‘early awakening’ and ‘morning depression’ as a proxy for the physical symptoms of depression, in a way less sensitive to confounding by antipsychotic side effects. Another conceptual difference is that the CDSS and other self-report questionnaires like the Center of Epidemiologic Studies-Depression [39], but not the QIDS-SR_16_, cover hopelessness. Patients with schizophrenia may be prone to psychological depressive symptoms like hopelessness and self-depreciation, possibly related to demoralization in response to the severe mental illness [40]. Thus careful item selection targeting only those depressive symptoms specific for patients with a psychotic disorder may be relevant for the validity of a self-report depression instrument in this population.

Although there was some overlap, the QIDS-SR_16_ discriminated depressive symptoms from negative symptoms in an acceptable way, in line with previous work on the full 30-item Inventory of Depressive Symptoms (IDS) in a mixed population of patients with schizophrenia and bipolar disorder [41]. In addition, a latent factor for negative symptoms was not identified for the QIDS-SR_16_, despite that several items overlap with negative symptoms, such as of concentration difficulties (question #10), lack of interest (#13) and lack of energy (#14). The current results suggest that, although the QIDS-SR_16_ may partly tap into the negative symptom dimension and thus should be interpreted with care, its divergent validity is acceptable in patients with psychotic disorders.

An unexpected result is the relatively high correlation of the CDSS with negative symptoms in comparison to previous reports of the CDSS in patients with schizophrenia [13]. Some correlation with negative symptoms is acceptable, as patients may often experience both negative and depressive symptoms at the same time [42]. Another caveat when interpreting the current results is that the majority of the patients had low EPS ratings. The relatively young and possibly well stabilized sample of patients may explain the rare presence of EPS, as previously described for the baseline measurement of the current sample [43]. We therefore remain inconclusive about the divergent validity of the QIDS-SR_16_ with respect to the extrapyramidal symptoms in this population.

An important strength of the study is its large sample size. A limitation of the study design may be that the same research assistant rated both the CDSS and the PANSS interview. This may have led to an overestimation of the correlation between the CDSS and the PANSS subscale for emotional distress, because of prior knowledge of the raters based on the previous interview. Therefore, the PANSS subscale for emotional distress does not necessarily outperform the QIDS-SR_16_ on its concurrent validity with the CDSS.

To conclude, we showed that patients with a psychotic disorder can reliably rate their depressive symptoms by means of the self-report. However, despite the fact that the QIDS-SR_16_ can provide clinicians with useful additional and clinically relevant information, we would not recommend applying the QIDS-SR_16_ for the assessment of depressive symptoms in this population, based on the poor concurrent validity of the QIDS-SR_16_ with the CDSS. Future research may focus on the development of a new self-report instrument, especially designed to assess depressive symptoms in patients with psychotic disorders.

## Conclusions

Seventeen percent of patients with psychotic disorders suffered from depressive symptoms. Although the Quick inventory of Depressive Symptoms (QIDS-SR_16_) may provide unique and clinically relevant information on depressive symptoms, this self-report instrument is not suitable for the use in patients with psychotic disorders. There is a need for a new self-reporting instrument covering depressive symptoms specific for patients with a psychotic disorder.

## Additional file

Additional file 1:
**The Quick Inventory of Depressive Symptomatology (16-Item) (Self-Report) (QIDS-SR**_**16.**_ **).**
